# Predictors of Communication Skills Following a Peer Partner Intervention for Young Autistic Children with Limited or No Spoken Language

**DOI:** 10.3390/bs16101962

**Published:** 2026-10-21

**Authors:** Kathy S. Bourque, Jessica R. Steinbrenner, Janet B. Weaver, Alexa M. Padak

**Affiliations:** 1 Juniper Gardens Children’s Project, University of Kansas, Kansas City, KS 66101, USA; janet.weaver@ku.edu (J.B.W.); alexapadak@ku.edu (A.M.P.); 2 Department of Health Sciences, University of North Carolina at Chapel Hill, Chapel Hill, NC 27599, USA; jessica.dykstra@unc.edu

**Keywords:** autism, social treatment, augmentative communication, predictors, children

## Abstract

Young autistic children with limited or no spoken language present with a myriad of social and communication challenges that restrict participation in school environments. Instructional approaches that include a speech-generating device (SGD) to augment speech and teaching peers to be supportive communication partners can lead to marked improvements in communication, expressive language, and social reciprocity. To inform clinical decisions and individualize approaches to meet child needs, more research is needed to identify key child-level behaviors that predict treatment response to social communication interventions. This study determined if pre-treatment levels of (1) expressive and receptive language, (2) nonverbal cognitive skills, (3) autism characteristics, and (4) peer-directed communication and reciprocity were related to child communication following an SGD intervention with peer partners. Participants included *N* = 56 autistic children (ages 3 to 6 years) with limited or no spoken language and *N* = 107 non-autistic peers (1–3 for each child). Peers learned to Stay–Play–Talk, then participated in 10 play-based intervention sessions with an autistic child implemented by trained school staff. An SGD with voice output app was provided for all children. Participants demonstrated significant increases in three social communication outcomes after intervention. Bivariate correlations were used to assess the relationship of potential predictors with these three key communication outcomes. Then, predictors with significant associations were included in multiple linear regressions to examine the contributions of child-level predictors for each outcome. All three regression models explained significant variance and had at least one significant child-level predictor (e.g., expressive language, autism characteristics, or nonverbal cognitive skills). Results provide support for identifying child characteristics that are precursors for growth in peer-directed social communication, and are thus important to teach to optimize outcomes for autistic children with significant communication challenges.

## 1. Introduction

Young autistic children with limited or no spoken language face significant challenges that impact social interactions, engagement in play, and overall participation in school activities. Approximately 30 to 40% will have persistent delays and remain nonverbal or minimally verbal (i.e., less than 20 spontaneous words) when they enter kindergarten (Tager-Flusberg & Kasari, 2013); thus, much more needs to be done to address their complex needs (Lord et al., 2022). Although progress has been made in identifying effective interventions for autistic children under five years (Smith & Iadarola, 2015), up to half of all autistic child participants have been characterized as slow or less than optimal responders to intervention (Eldevik et al., 2010). One highly researched and effective intervention is teaching children to use Augmentative and Alternative Communication (AAC), with wide acceptance of iPads or mobile devices as speech-generating devices (SGDs) with the installation of voice output apps (Wendt, 2024). Within natural preschool activities, preschoolers learning to use AAC show significant increases in communication and reciprocal exchanges with peers who are taught to be responsive in play (Bourque, 2020; Therrien et al., 2016). Currently, there is limited research identifying child-level behaviors or characteristics (e.g., communication, social skills) that may be associated with response to AAC interventions, including when AAC is combined with peer support approaches. Understanding why early interventions may be more appropriate for some autistic children with limited language and less effective for others is important to individualize or adapt approaches to meet children’s needs.

### 1.1. AAC and Communication Partner Instruction for Young Autistic Children

There are decades of empirical support demonstrating that teaching young autistic children to use AAC can improve a wide range of communication skills, including gestures, expressive and receptive vocabulary, and spoken words, as measured through direct observations and standardized language tests (see reviews by Privitera et al., 2026; Schlosser & Koul, 2015). Interventions often included blended approaches from established Naturalistic Developmental Behavioral Interventions such as Enhanced Milieu Teaching (EMT; Kaiser & Hampton, 2017) and Joint Attention, Symbolic Play, Engagement and Regulation (JASPER; Kasari et al., 2021a) with a focus on functional communication and natural social reinforcers. Within EMT, naturalistic strategies include environmental arrangement, following the child’s lead, responding contingently, language expansions, time delay, and supportive prompts to elicit communication. JASPER strategies include environmental arrangement, expanding on nonverbal and spoken communication within play routines, language modeling, creating opportunities for joint attention and requesting, and supporting children’s regulation. Kasari et al. (2014) observed greater spontaneous communication and language outcomes for 61 autistic elementary-age children (5 to 8 years) with limited language when adult SGD input was provided within a blended JASPER and EMT intervention compared to children who did not receive SGD models. Following this seminal study conducted in clinic playrooms, researchers recognized the need to examine the impact of blended interventions in authentic environments for autistic children learning to use AAC.

A child’s early social communication development is accelerated by interactions and changes in their social environment. When engagement is viewed as a bidirectional interaction (Chen et al., 2021), it becomes clear that teaching all communication partners is essential. Using an ecobehavioral assessment, Hume et al. (2019) examined patterns of social interactions for 52 autistic preschoolers in inclusive classrooms. They found that children engaged with peers very rarely (3% of intervals observed), and when they were engaged adults were not present. This finding supports the recommendation over years of AAC research that aided communication should start as early as possible, with peer partner instruction as a necessary component to enhance social communication and classroom engagement (Kent-Walsh et al., 2015; Romski et al., 2015). Over the years, supporting non-autistic peers within social interventions has been described in a number of ways. These include peer-mediated interventions, peer training, peer support strategies, peer modeling, and communication partner instruction when supporting AAC users (Kent-Walsh & McNaughton, 2005). The term communication partner instruction is used throughout this paper to emphasize a partnership-based approach, rather than positioning peers as agents of intervention for autistic children.

Thiemann-Bourque et al. (2018) developed and examined a modified Stay–Play–Talk (SPT) intervention (English et al., 1997; Goldstein et al., 1997) that added SGD instruction during the *Talk* phase. The original *Stay* (i.e., maintain proximity), *Play* (i.e., say name, suggest a play activity, make comments) and *Talk* (i.e., keep playing and talking to your buddy) intervention was developed to provide a simple set of strategies that required minimal effort for peers to learn and for teachers to implement in inclusive preschool settings. Thiemann-Bourque et al. (2018) conducted a randomized controlled trial (RCT) with 45 autistic preschoolers with limited or no spoken language, 95 non-autistic peers (1–3 for each child), and 42 school team members. Autistic children were provided with an iPad as SGD to use for the school year. Children were randomly assigned to SPT + SGD or SGD only (i.e., staff instructed to use SGD as they deemed appropriate within play with peer). In the experimental group, peers were taught to *Stay* (e.g., sit close), *Play* (e.g., share toys), and *Talk* (e.g., look and listen, say words out loud) prior to participating in weekly intervention sessions with their autistic partner. Post-treatment results demonstrated that when SGD instruction was combined with SPT, children’s spontaneous communication to peers was significantly higher compared to children in the SGD only group. Results also showed more balanced reciprocity (50:50 child-to-peer acts) during treatment and generalization settings for children receiving SPT and SGD. The authors observed high fidelity of staff implementation (i.e., average 89%), and peer implementation (i.e., average 80%). Together, these positive outcomes support the current study rationale to examine predictors of treatment response for young autistic children following a peer partner intervention that could be feasibly implemented by early service providers. In addition, while the SPT + SGD approach was more effective overall, there were individual differences in communication outcomes within both groups, highlighting the heterogeneity in how autistic children learn and the need to examine child behaviors that predict their response to AAC interventions.

### 1.2. Language and Reciprocity as Predictors of Communication Intervention Response

Due to the wide range of behavioral characteristics and varying developmental trajectories of autistic children, selecting intervention approaches and identifying communication and social skill targets can be challenging. Furthermore, not all children respond similarly to the same intervention. Kasari et al. (2021b) expressed the need for an evidence-based approach to intervention, “…that simultaneously (a) provides interventions that have been shown to accelerate the development of social and academic engagement and (b) adapts such interventions to the changing needs of the child (e.g., if there are indications of slow response)” (p. 2). To better understand why one type of AAC or communication partner intervention may work for one child but not for another, or why an intervention that worked in the past does not work in the future for the same child, more research is needed to identify child-level behaviors that relate their response to these approaches. If specific child behaviors are found to be related to lower social communication performance with peers after intervention, then it may be possible to identify other approaches that need to be instituted first (e.g., those that focus on improving the social behaviors themselves) and tailored to match child strengths and learning needs.

For young autistic children in general, research suggests that language and cognitive ability are consistent predictors of treatment response, as those with higher pre-treatment language and cognitive skills often respond better (Vivanti et al., 2014). In a recent large study with 157 autistic children (ages 33 month to 54 months) with global developmental delays and limited language, Kasari et al. (2023) compared two different early evidence-based early intervention models found to improve social communication and spoken language, discrete trial training (DTT) and JASPER (Kasari et al., 2021a). The DTT approach emphasized adult-led teaching of differential responses to stimuli and systematic reinforcement to improve skills such as imitation, play, matching, and requesting. The authors reported that expressive language improved for children receiving either intervention; however, children with stronger receptive language and joint attention skills responded more from JASPER. In an exploratory study with 24 autistic preschoolers with limited or no spoken language, Bourque et al. (2024) also found that children with higher receptive language at the start showed greater gains in peer-directed expressive language (i.e., SGD and speech) and reciprocal turns in dyadic play following participation in a Stay–Play–Talk intervention. The authors also reported significant correlations between pre-treatment expressive language scores on a standardized test and changes in children’s total communication (i.e., SGD, speech, SGD + speech, gestures, and vocalizations) post-treatment with peers in a free-play classroom setting. This preliminary finding aligns with research suggesting that the ability to express even one word or imitate speech may lead to stronger child outcomes (Tager-Flusberg & Kasari, 2013). At this time, much more research is needed to understand what child characteristics relate to positive social communication outcomes for young autistic children with limited or no spoken language.

Participating in and maintaining reciprocal interactions is a core challenge for autistic children (American Psychiatric Association, 2013) and may be important to consider as both a predictor and outcome of communication interventions. Locke et al. (2016) found that elementary-aged autistic children spend almost half the time jointly engaged in reciprocal social activities compared to non-autistic classmates. As a treatment outcome, DiStefano et al. (2016) examined the length and number of exchanges, and child-initiated turns for minimally verbal autistic children ages 5–8 years during interactions with adult partners in a clinic setting. They found that growth in reciprocal communication was observed only after SGD modeling (i.e., SGD use paired with a minimum of 50% of adult spoken language acts) was added to the blended 6-month JASP + EMT intervention and the number of exchanges a child participated in prior to treatment was positively related to progress in amount of spoken language gained after treatment.

In school contexts, children’s ability to maintain back-and-forth interactions with peers is critically important, as successful balanced exchanges may lead to higher quality interactions (Goldstein & Kaczmarek, 1992). Thiemann-Bourque and colleagues expanded knowledge on the impact of SGD interventions on reciprocity by measuring autistic children’s and peer communication behaviors (Thiemann-Bourque et al., 2017, 2018). When peers were taught to be responsive communication partners, outcomes showed marked increases in reciprocal interactions (i.e., communicating within 3 s of the previous communicative act) between autistic preschoolers learning to use AAC and peers in table-top play (e.g., cause–effect toys, puzzles, and simple games) and snack time. If children can take turns and maintain longer communication exchanges, they may have more opportunities to observe and learn from peer models. Competencies in early social and language skills provide a foundation for later peer relationships and academic achievement. For example, in a recent longitudinal study of 43 autistic individuals assessed as toddlers, Saban-Bezalel et al. (2025) found that the group with greater improvements in social communication formed more friendships in adolescence. Further, stronger expressive language in autistic toddlers has been reported to predict higher reading comprehension skills at kindergarten entry (Davidson & Ellis Weismer, 2014).

### 1.3. Considerations for Measuring Early Social Communication

Early prelinguistic skills (e.g., gestures, vocalizations) are important to examine as potential predictors of treatment response because they serve as foundational behaviors that may support children’s responsiveness to social communication interventions (Laister et al., 2021). Over the first two years, non-autistic children become more adept at using a range of communication modalities, moving from prelinguistic to linguistic or symbolic communication. The development of behaviors such as giving, pointing, and combining sounds to vocalize indicates more complex presymbolic communicative modes. These early skills, along with other social competence behaviors (e.g., joint attention, imitation, social interest/engagement), are closely associated with and provide a foundation for later language, peer relationships, and learning readiness (Alzahrani et al., 2019; National Autism Center, 2015; Warreyn et al., 2014).

For young nonverbal autistic children, assessment of an array of communication modalities is essential to understand unique developmental profiles and strengths that may be important for subsequent engagement and communicative interactions with peers. Koegel et al. (2020) reviewed research on evidence-based assessments for minimally verbal and verbal autistic individuals and concluded that very few studies assessed prelinguistic behaviors as predictors of communication abilities. We addressed this gap in the literature by including spontaneous gestures and potentially communicative vocalizations (i.e., evidence of social orientation to the joint activity or to the peer) as behaviors in a ‘total communication’ variable that also included intentional speech and SGD use. Furthermore, examining rates of child communication during naturalistic play observations as a potential predictor of communication outcomes provides another context other than standardized testing that alone may not provide satisfactory information. That is, standardized assessments with young autistic children are often unreliable due to limited representation in the standardized sample (Zimmerman et al., 2011), floor effects, the expectation for social compliance, behavioral challenges, and a lack of credit for responding with AAC systems.

### 1.4. Present Study

In summary, these results support expressive language, receptive language, and joint attention as predictors of treatment response for young autistic children who are not yet talking or talking very little. In addition, notable relationships have been reported between early social competencies and later peer interaction skills. Measuring proximal behaviors such as expressive and receptive language skills moves beyond prior efforts focusing on more global child variables (e.g., cognitive skills; Vivanti et al., 2014). Assessing children’s baseline levels of peer-directed communication as potential predictors is also of interest as these are malleable behaviors that could be selected as intervention targets. Thus, outcomes may allow for identification of early child language and social communication characteristics related to treatment response, and the need to tailor approaches to support children’s social development. The purpose of the present study was to expand knowledge on potential predictors of response to a peer communication partner intervention for young autistic children in preschool and kindergarten who are learning to communicate with an SGD. The research aims included: (1) What are the effects of a Stay–Play–Talk with SGD peer-partner intervention on spontaneous communication, reciprocal turns, and reciprocal exchange length of young autistic children with limited or no spoken language? (2) What child-level characteristics predict communication, reciprocal turns, and reciprocal exchange length with peer partners after intervention?

## 2. Methods

### 2.1. Participants

Participants included 56 autistic children between the ages of 3.1 and 6.0 years (mean age 4.3); 84% of the participants were male. The children represented diverse racial backgrounds (i.e., 48% White, 32% Black, 14% Multiracial, 4% American Indian/Alaska Native, and 2% Asian). Over a four-year period, children were recruited from preschool and elementary schools in the Kansas City metro area in Kansas and Missouri, Durham-Chapel Hill metro area in North Carolina, and the Atlanta metro area in Georgia. To be eligible, children met the following inclusion criteria: (a) confirmed (*n* = 46) or suspected (*n* = 10) autism diagnosis and confirmation of autism characteristics on the Autism Diagnostic Observation Schedule–Second Edition (ADOS-2; Lord et al., 2012) administered by research staff, (b) limited or no spoken language defined by less than 20 functional spontaneous words, (c) a candidate for a speech-generating device (SGD) or already using one, (d) access to non-autistic peers at school, and (e) English as a primary language spoken at home (i.e., one caregiver spoke English with proficiency to understand and express 50% of communication). Limited or no spoken language was confirmed based on parent and teacher report, and standardized language testing. Any children observed to produce more than 20 spontaneous, non-scripted words were not included. Children were also excluded if they had physical or motor impairments that prevented selection of SGD icons and/or a lack of symbol discrimination skills as determined by the school team.

Three standardized assessments were administered at the autistic child’s home or school within one month of the start of observations to confirm eligibility and to measure baseline child-level language and social behaviors for the analyses. These included (1) the ADOS-2 Module 1 (Lord et al., 2012), (2) the Mullen Scales of Early Learning (MSEL; Mullen, 1995), and (3) the Preschool Language Scale–Fifth Edition (PLS-5; Zimmerman et al., 2011). The ADOS-2 administrators were research-reliable, and the first and second authors were trained research assistants on the PLS-5 and the MSEL (see Table 1 for demographic information).

Non-autistic peers (*N* = 107) were recruited from the same school program as the autistic child. One to three peers (41 males and 66 females; mean age 4.8 years of age) were paired up with each autistic participant. School staff recommended peer participants who would be responsive models based on their ability to: follow simple directions, take turns, offer to help other children, and have consistent school attendance. One or more school staff participants (*N* = 44) were recruited from each autistic child’s school team with a wide range of early service providers (e.g., speech-language pathologists, special education teachers, paraprofessionals, and behavior therapists) learning to implement the intervention. Some implementers participated with multiple children over different school years.

This study was approved by the Human Research Protection Program at the University of Kansas and all ethical considerations for protection of participants were followed. Research staff obtained parental consent for all children prior to the start of study, and verbal assent from non-autistic peers before the first observation. All school staff consented to participate.

### 2.2. Study Design

This study used a one-group pretest–posttest design for 56 autistic children and 107 non-autistic peers. All participants received the *Stay–Play–Talk* intervention, with each autistic child paired with 1–3 peer partners; multiple peers took turns rotating into the group every other week. All children received 10, 30 min intervention sessions twice per week for approximately 5 weeks. Data collected are part of a larger Sequential Multiple Assignment Randomized Trial (SMART) investigating a Stay–Play–Talk and SGD intervention with the same participants. The larger study examines different intervention features implemented over a longer period of time (i.e., up to 24 sessions), which will be reported separately at the end of that study to avoid experimenter bias. Importantly, all participants in this study received the SPT intervention with a speech-generating device.

### 2.3. Measures

#### 2.3.1. Standardized Assessments

Child performance on three standardized assessments administered prior to the start of intervention are used in our analyses. We included children’s raw scores on two domains of the ADOS-2 Module 1: Social Affect (SA) and Restricted and Repetitive Behavior (RRB; Lord et al., 2012). The ADOS-2 is considered a gold-standard observational tool for assessing individuals with autism (Bölte et al., 2011). It includes a range of play-based tasks and activities to elicit communication, social interaction, and play skills. As potential language predictors, we included children’s performance on the Expressive Communication (EC) and Auditory Comprehension (AC) subtests from the Preschool Language Scale-5 (PLS-5; Zimmerman et al., 2011). The PLS-5 is a norm-referenced, play-based language assessment for children from birth to 7 years, with a range of items that test receptive and expressive language and allow for parent-report on earlier skills. Examples of receptive items include: responds to inhibitory word (e.g., “no”), understands one word/phrase without gestural cues, demonstrates functional and relational play, and follows directions with gestural cues. Early expressive items include: takes two turns vocalizing with caregiver, plays simple games with eye contact, vocalizes different consonant sounds, and uses symbolic gestures (e.g., waves, claps hands). From the Mullen Scales of Early Learning (MSEL; Mullen, 1995), we included children’s performance on the Visual Reception (VR) and Fine Motor (FM) subtests as a measure of nonverbal cognitive skills and motor planning. The VR items assess children’s ability to process and interpret visual information (e.g., shapes, patterns, spatial organization) and is predictive of later nonverbal cognitive development (Caudle et al., 2014). Both subtests reduce speech and language demands, separating expressive and receptive language from nonverbal abilities. For both the PLS-5 and the MSEL subtests, we included raw scores due to floor effects across participants (i.e., standard scores were at the lowest possible value) and low variability.

#### 2.3.2. Coding of Communication Behaviors

Communication outcomes were coded from the video-recorded child–peer play sessions collected after 10 sessions (i.e., not intervention sessions). Both the autistic child and peer spontaneous communication acts were coded during the 10 min observations using Noldus Observer XT (v17) software (Noldus Information Technology, 2023; Leesburg, VA, USA). The Noldus behavior coding scheme was developed in the first author’s prior RCT study examining a similar intervention and population of autistic preschoolers (Thiemann-Bourque et al., 2018). Data was averaged across the two 10 min observations (i.e., 20 min total) and converted to a rate per minute of all communication acts. To be coded, each child communication act had to meet the requirements of being spontaneous and intentional; that is, peer or adult prompts to the autistic child were not coded and all acts were directed towards the other child or to the shared activity to show orientation and joint engagement. Five communication modalities were coded: (1) speech (e.g., words or word approximations), (2) SGD use (e.g., selects symbol on iPad), (3) speech combined with SGD (e.g., spoken language and SGD within 3 s of one modality), (4) vocalizations (e.g., potentially communicative sounds or sound combinations), and (5) gestures (e.g., reach, head-shake, or signs) (see Table 2). Each communication act was coded as an initiation or a response, with initiations coded based on who started to communicate and responses coded if the other partner responded within 3 s of the last response. Thus, sequences of consecutive responses could be coded if there were less than 3 s between each child’s communication act. If more than 3 s passed, the next communication act was coded as a new initiation. No communication directed to adults was coded.

From the video-coded data, we calculated one measure of child communication and two measures of reciprocal communication. Child communication was the total number of spontaneous communication acts per minute, which included all five communicative modalities. Reciprocity was defined based on when one child initiated and the other child responded within 3 s, with each partner taking a minimum of one turn to be considered a reciprocal exchange. The length of the exchange continued if either child responded within 3 s of the prior communication act and continued until there was a new initiation (i.e., minimum 3 s pause). The two reciprocal communication measures were: (1) number of communicative turns per minute (i.e., labeled as reciprocity) and (2) number of communicative turns within each exchange (i.e., labeled as exchange length). Reciprocity was calculated based on the rate of turns per minute across the 20 min of video data. Exchange length was calculated based on summing the total number of turns taken and dividing by total number of reciprocal exchanges across the 20 min of video data.

### 2.4. Settings and Materials

All observations, peer instruction, and intervention sessions took place at preschool and elementary schools in a quiet location (e.g., separate room or behind a partition in a classroom) recommended by the school team. Research staff brought a container of toys for implementers to use based on a parent and school staff report of child preferences, with new toys rotated in after 2–3 weeks to maintain child interest. School staff were encouraged to use their own toys when possible. Activities included puzzles, sensory toys (e.g., bubbles, pop rockets, squishy balls), cause–effect toys (e.g., light up gear toy, stacking wheel spin stick, and piggy bank), ball toys (e.g., ball popper, pound a ball, and gumball), manipulatives (e.g., play-doh, peg boards, car racer), pretend play (e.g., pizza party, ice cream, magnetic animals), and simple games (e.g., Pop-up Pirate).

### 2.5. Procedures

#### 2.5.1. School Staff Training and Ongoing Support

Training and ongoing support were provided to school staff by the research staff. All research staff were trained by the first and third author using teaching scripts and video examples from our prior SPT preschool intervention studies or from the current study. They were supervised and independently led sessions or coached school staff after completing the training protocol.

Prior to baseline observations, school staff implementers attended a 1 h introductory training on: (1) study goals and timeline, (2) peer recruitment, (3) setting up child-preferred activities, and (4) programming the SGD. The autistic children received an iPad programmed with a voice output app called TouchChat^®^ WordPower (v2.0) (PRC-Saltillo, 2026b; Wooster, OH, USA) for the duration of the study. If a child was already using a different voice output app (e.g., LAMP Words for Life^®^ v2.0) (PRC-Saltillo, 2026a; Wooster, OH, USA), they continued to use that app. Vocabulary pages on the SGD provided a variety of reasons to communicate, and each page was programmed with a minimum of four communicative functions: (1) requests for objects or actions, (2) comments, (3) protests, and (4) gaining attention (i.e., name and photos of children). The number of vocabulary symbols on a page ranged from 20 to 84 across participants.

Following baseline observations, school staff completed an additional 1 h training focused on: (1) implementer expectations (e.g., familiarity with SGD, giving full access to the device), (2) peer-partner skills to teach, and (3) intervention materials with video examples demonstrating steps (e.g., peer review, child–peer practice, how to prompt and provide feedback). Each implementer received a binder with a peer-partner instruction script, child–peer intervention script, and two prompt cards (one for adult and one for peer) as visual reminders to use during sessions.

As part of ongoing support, research staff implemented up to three intervention sessions (described below) and school staff followed along with their script. After the first three sessions, research staff transitioned to a coaching role to guide and provide feedback on following the script, creating communication opportunities, and switching toys out if needed. Coaching of school staff occurred approximately every other week.

#### 2.5.2. Peer Partner Communication Instruction

All peers were taught to be responsive communication partners during two 30 min sessions; autistic children did not attend these sessions. Research staff led the instruction and school implementers observed and participated in role-play activities. A Buddy Book with cartoon pictures depicting children engaging in the Stay–Play–Talk steps was used to teach peers to: (1) Stay—sit close, if your buddy moves you move; (2) Play—share toys, take turns; and (3) Talk with your Buddy—say a name, say what you see, and ask something. For each step, instruction followed a standard protocol of labeling and describing each step, modeling and role-play of each step, and giving feedback. A laminated 5 × 7 card with the cartoon pictures of each step was introduced and used during intervention as a visual reminder.

#### 2.5.3. Child–Peer Intervention Sessions

After the peer partner instruction, the autistic child joined one peer for 20 to 30 min sessions, twice per week for 10 sessions; multiple peers participated on a rotating basis. Each session consisted of: (1) 5 min adult–peer review of all SPT steps (only peer present), (2) 5 min adult-guided peer practice of steps with autistic child present, (3) 10 min child–peer play, and (4) 1–2 min adult feedback (i.e., one strength and one skill for peer to work on next time). During the 10 min child–peer play, the implementer listened for communication from either child, then used a least-to-most prompting hierarchy to encourage communication. School staff were encouraged to download a free phone app that would alert them to prompt approximately every 30 s throughout the 10 min. An example of the prompting hierarchy was (1) “Remember to talk out loud”, if peer did not respond follow up with (2) “Say *[child’s name]*, what do you want?”, and then (3) provide support to help autistic child communicate (e.g., point to icon “You can push this and say____”).

#### 2.5.4. Pre- and Post-Treatment Video Data Collection

All child–peer observations were video-recorded using a Panasonic or Sony camcorder on a tripod set up close to the table. Recordings were then uploaded to a secure server in the research lab to be coded with Noldus Observer at a later time. Pre-treatment video data was collected prior to peer-partner instruction over two sessions on two different days, for a total of 20 min. Post-treatment video data was collected after the 10 child–peer intervention sessions, over two days for a total of 20 min. During these sessions, research staff instructed the children, “*You’re going to stay and play at a table for 10 min. I have different toys. I can show you how one works if you need help. I’m going to watch today.*” Within each session, each activity was presented one at a time and children were encouraged to play for 2 min (at minimum) before transitioning to a new toy. The child’s iPad was placed on the table, and the staff selected one vocabulary page that matched the activity. Research staff did not provide prompts and only intervened to manage challenging behaviors (e.g., “Let’s play nicely”) and to transition between toys.

### 2.6. Treatment Fidelity and Social Validity

Fidelity of adult implementation and peer implementation was randomly checked for 33% of treatment sessions after initial modeling by research staff (i.e., for treatment sessions 4 through 10, completed a total of 128 of 392 sessions). Trained research staff completed cross-site fidelity checks (i.e., KU, UNC, and Emory locations) to prevent the primary coaches from reviewing videos of same-site school staff implementers. For the adult fidelity of implementation, the average was 89% (range from 53 to 100%), and the average peer fidelity was 90% (range from 50 to 100%).

After 10 intervention sessions, implementers completed a three-item social validity survey administered electronically via REDCap. Implementers rated autistic children’s improvement in level of engagement, frequency of communication to peer, and the quality of child–peer interaction during play using a 1–3 Likert scale: 1 (Not at all), 2 (Somewhat), and 3 (Very much). The mean implementer rating across *N* = 43 autistic children was 2.4 for each question, which relates to *Somewhat to Very much*.

### 2.7. Data Analytic Plan

All data analyses were conducted using JMP Student Edition Version 19.1.0 (JMP Statistical Discovery LLC, 2026). We first examined descriptive statistics including mean, standard deviations, and skewness for predictor and outcome variables. We also examined visual data to assess the presence of outliers. For the ADOS-2, we examined the algorithm total scores for the Social Affect (ADOS-SA) and Restricted and Repetitive Behaviors (ADOS-RRB) to be able to look at the domains separately. The scores were relatively normally distributed. For the PLS-5 (AC and EC) and MSEL (VR and FM) subscales, we used raw scores due to floor effects on standardized scores. The raw scores were relatively normally distributed in the sample. Data for video-coded communication, reciprocity, and exchange length were examined separately for predictor (baseline) and outcome (post-treatment) variables. Across video-coded variables, the data were generally positively skewed (more observations at lower values) and included some outliers (between two and four participants per variable above two standard deviations) and determined that data were sufficiently normally distributed for multiple linear regression analyses.

To address our research aims, we examined descriptive statistics for the three video-coded outcomes (communication, reciprocity, and exchange length). We also conducted Wilcoxon Signed-Rank Tests (based on non-normal distribution of difference scores) to determine if there were significant changes from baseline to post-treatment (i.e., the 10-session intervention mark). Then, we examined bivariate correlations between potential predictors (ADOS-SA, ADOS-RRB, PLS-5 AC raw score, PLS-5 EC raw score, MSEL-FM raw score, and MSEL-VR raw score; raw scores used due to floor effects and low variability in standard scores) and outcomes to determine which predictors to include in the final regression models for each of the three outcomes. We also visually inspected bivariate scatterplots to assess if relationships appeared linear in nature.

Next, we conducted multiple linear regression analyses for each of the three outcomes, including baseline scores of the outcome and relevant predictor variables. Following regression analyses, we examined the normality of residuals and homoscedasticity and assessed influential cases through Cook’s D and Studentized Residuals. Although most values were within acceptable limits, one participant had Studentized Residuals over the recommended value of three in all three regression models. Although the participant’s scores were within expected ranges for the sample population, the results of the multiple linear regression were different when the participant was removed from the sample. Thus, we report the multiple linear regression results removing the single participant with Studentized Residuals over threshold across all three models below but provide results with the full sample in Supplementary Materials.

#### Interobserver Agreement

Interobserver agreement (IOA) was completed for total communication acts, initiations, responses, and communication behaviors (i.e., vocalizations, gestures, SGD, speech, speech + SGD) for autistic children and peers. All observations were coded by research assistants who were not involved in providing intervention for dyads coded and trained by the first author. To become reliable, coders had to reach 80% inter-rater reliability on three gold-standard videos (from the first authors prior studies with similar population and social dyads) for all communication categories coded for the autistic children and peers. During training, inter-rater reliability was determined point-by-point; that is, an agreement was coded if both primary and reliability coder observed and agreed on a communication act, within 10 s. Time stamps within Noldus Observer guided calculations for timing inter-rater reliability.

From baseline and post-treatment observations, a minimum of 25% were coded by a secondary or reliability coder blind to treatment procedures. For all sessions, data was exported from Noldus into excel for analysis. Reliability was evaluated using Intraclass Correlation Coefficients (ICCs) calculated in IBM SPSS (Version 31.0). Following the recommendations by Koo and Li (2016), a two-way mixed effects model was used, in which participants were treated as random effects and coders as fixed effects. The ICCs were calculated based on absolute agreement for the following: autistic child initiations, autistic child responses, autistic child behaviors, peer initiations, peer responses, and peer behaviors. All ICCs were above 0.80, with baseline ranging from 0.90 to 0.97, and post-treatment ranging from 0.80 to 0.99. ICC values between 0.75 and 0.90 indicated moderate reliability and values greater than 0.90 indicating excellent reliability.

## 3. Results

### 3.1. Differences Between Outcomes from Pre-Treatment to Post-Treatment

The video-coded data included communication (i.e., rate of all spontaneous communication acts), reciprocity (i.e., rate of turns per minute), and exchange length (i.e., mean turns per exchange). The mean rate of spontaneous communication acts was 0.84 acts per minute at baseline (just under 17 acts in the 20 min of video on average) and 1.93 acts per minute after 10 intervention sessions (over 38 acts on average in the 20 min of video), showing significant change in a Wilcoxon Signed-Rank Test (*S* = 677.50, *z* = 0.87 *p* < 0.0001). The mean rate of turns changed from 0.30 per minute (about 6 turns in the 20 min of video) to 1.25 per minute (25 turns in 20 min of video on average), also showing significant growth (*S* = 719.50, *z* = 0.91, *p* < 0.0001). Finally, the mean turns per exchange were 1.02 at baseline and 1.56 after 10 intervention sessions, with significant growth from baseline to post-treatment (*S* = 690.50, *z* = 0.77, *p* < 0.0001). Effect sizes were calculated using r (absolute value of z score divided by square root of n) and were small with values of 0.12, 0.12, and 0.10 for rate of communication, rate of turns, and turns per exchange, respectively. See Table 3 for additional descriptive data and full Wilcoxon Signed-Rank Test results.

### 3.2. Correlations Between Potential Predictors and Outcomes

Since communication, reciprocity, and exchange length showed significant growth between time points, all outcomes were included in predictor analyses. Based on the exploratory nature of this study, we used a data-driven approach to determine which predictors should be included in each model. First, we examined correlations between the outcomes and potential predictors, which included measures of autism symptomatology (ADOS-2 SA and ADOS-2 RRB), language (PLS-5 AC and PLS-5 EC), and other developmental measures for Visual Reception and Fine Motor skills (MSEL VR and MSEL FM; see Table 4). Communication was significantly correlated with three standardized measures (ADOS-2 SA, PLS-5 EC, and MSEL VR) and two of the video-coded measures (baseline communication and baseline reciprocity). Reciprocity was significantly correlated with four standardized measures (ADOS-2 SA, PLS-5 EC, MSEL VR, and MSEL FM) and two video-coded measures (baseline communication and baseline reciprocity). Exchange length was significantly correlated with two standardized measures (PLS-5 EC and MSEL VR) and all three video-coded measures (baseline communication, baseline reciprocity, and baseline exchange length). We examined correlations between the significant predictors to assess multicollinearity. There were strong correlations in the area of concern between MSEL VR and MSEL FM (r = 0.84) and baseline communication and reciprocity (r = 0.71). Based on this, Mullen FM was not included in the regression model for reciprocity, and either baseline communication or baseline reciprocity was selected for inclusion in each of the three models based on the highest correlation with the outcome variable.

### 3.3. Multiple Linear Regression Models with Predictors and Communication Outcomes

The regression model for communication included baseline communication, ADOS-2 SA, PLS-5 EC, and MSEL VR. The overall model was significant (*F* = 9.06) and explained 42% of the variance (see Table 5). Baseline communication, PLS-5 EC, and ADOS-SA were all significant predictors in the model. Each additional point of baseline rate of communication acts is associated with a 0.70-point increase in the 10-session post-treatment rate of communication acts. Each additional point on the PLS-5 EC raw score is associated with a 0.07 increase in rate of communication acts (just under one additional act per 10 min) and each additional point on the ADOS-2 SA algorithm score is associated with a drop of 0.08 in rate of communication acts (just under one fewer acts per 10 min). Based on the standardized coefficients, the baseline scores were the largest contributor, followed by the PLS-5 EC and then the ADOS-2 SA. The MSEL VR raw score contributed little compared to the other variables.

For reciprocity (i.e., number of reciprocal turns), the predictors included baseline reciprocity, ADOS-2 SA, PLS-5 EC, and MSEL VR. Again, the overall regression model was significant (*F* = 8.84) and explained 41% of the variance (see Table 6). Only the baseline score of reciprocity was significantly predictive of reciprocity outcomes such that an increase in 1.0 turn per minute is associated with a 1.2 increase in the same metric post-intervention. This suggests that, overall, children with higher baseline reciprocity tend to have higher reciprocity after intervention. The contribution of the baseline PLS-5 EC score trended towards statistical significance (*p* = 0.056). The standardized coefficients indicate that the baseline reciprocity score was the greatest contributor to variance, and then PLS-5 EC and ADOS-2 SA were not significant contributors based on the model, but the standardized beta indicated that the variables were comparable at the level of contribution. Again, the MSEL VR score had relatively little contribution.

The predictors for exchange length included baseline exchange length, baseline reciprocity, PLS-5 EC, and MSEL VR. The overall model was significant (*F* = 7.05) and explained 36% of the variance (see Table 7). Both baseline exchange length and the PLS-5 EC raw score were significant predictors of exchange length at post-treatment. A one-point increase in the raw score of the PLS-5 EC is associated with an increase in 0.03 turns per exchange. Relatively speaking, baseline exchange length contributed the most, followed by the PLS-EC raw score. The baseline reciprocity score was not a significant contributor, though still had a modest standardized beta value. Similar to other models, the MSEL VR score contributed little relative to other included predictors.

## 4. Discussion

The results of this study are noteworthy and add to the limited research currently available examining AAC interventions and predictors of communication response for young autistic children with limited or no spoken language during social play with non-autistic peer partners. We evaluated video-coded observational data from child–peer play sessions for 56 autistic preschool and kindergarten-age children and 107 non-autistic peers, as well as children’s performance on standardized tests, to determine what child characteristics predicted spontaneous communication with peers following a 10-session social intervention. Our findings show that the predictors in each regression model contribute a significant amount of variance in post-treatment communication with peers, with each model identifying at least one significant child-level predictor. Two baseline Expressive Communication measures predicted the rate of communication and exchange length, but not reciprocity; baseline reciprocity was the only predictor of reciprocity post-treatment. Outcomes also reinforce findings from a resurgence of studies examining *Stay–Play–Talk* (Osborne et al., 2019; Severini et al., 2019), in that combining this approach with SGD instruction led to improved spontaneous communication in play with peer partners after a relatively short treatment period.

Across each of the models, the baseline score of the same metric as the outcome was the strongest predictor of post-treatment communication-related outcomes with peer partners. That is, children who communicated more often and participated in more or longer reciprocal exchanges with peers before intervention engaged in higher communication behaviors with peers after intervention. This finding has important clinical implications for young children with limited communication skills who are learning to use AAC. First, although not causative, the associations between pre-intervention social communication competencies in play with peers and post-treatment competencies clearly suggest the need to monitor how often autistic children are independently communicating with peers and if they are able to sustain interactions (e.g., either by initiating or responding to peer bids for attention). Children entering preschool or kindergarten with lower communication skills may require additional supports, such as a longer period of intervention or more time in 1:1 adult-guided AAC instruction, to support meaningful gains in peer-directed communication. Furthermore, outcomes reinforce the recommendation to evaluate a range of early communication modalities (e.g., vocalizations and gestures) as possible precursors to successful social communicative interactions for young nonverbal or minimally verbal autistic children (Koegel et al., 2020). For autistic children with stronger communication, intervention goals may need to be tailored to teach more complex social communication skills across peers, school contexts, and play routines rather than early social engagement skills (e.g., playing with, orienting to, and observing peers).

The measures of reciprocity and exchange length as predictor and outcome variables are based on bidirectional interactions between the autistic child and non-autistic peer partners. Although our child–peer observations were in semi-structured play, the minimal number of turns (e.g., 6 turns in 20 min of play) and length of turns (e.g., 1 turn per exchange) at baseline strengthen reports of limited engagement with peers when adults are not engaged (Hume et al., 2019). Analyses revealed that the initial ability to take reciprocal turns (i.e., to initiate or respond) with a peer was the only significant predictor of taking communicative turns in play after intervention. Furthermore, children’s ability to participate in longer back-and-forth exchanges and their standardized expressive language score at baseline significantly predicted their turns within exchanges after intervention. Interestingly, the children’s Visual Reception performance on the MSEL, often included in research as a measure of nonverbal cognitive skills, was not significantly related to any communication outcome and added little predictive value in all models. This finding suggests that Visual Reception as measured by the Mullen (i.e., matching objects, shape puzzles, sorting objects by shape, size, or color) may be less useful than understanding children’s existing expressive language and ability to participate in communication exchanges with peers as characteristics related to response to this SGD intervention. By including two child-level reciprocity behaviors in our analyses, this study addresses an important gap in social intervention research and highlights the value of measuring reciprocal social communication between autistic children with complex communication needs and their peers. The improved reciprocity outcomes following a brief AAC intervention underscore the importance of teaching children to initiate and respond, within peer social interactions and play routines.

As alluded to above, the predictive value of standardized scores were lower relative to the measures gathered from child–peer play sessions using observational coding. Receptive language (i.e., PLS-5 AC) did not exhibit significant bivariate correlations with any of the outcomes, thus did not warrant inclusion in the larger models. This differs from previous research that has suggested receptive language may be a meaningful predictor of outcomes in autistic children who are minimally verbal or non-speaking (Bourque et al., 2024; Kasari et al., 2021b). These contradictory findings warrant further investigation to determine if there is something unique about the sample or outcomes in the current study that may have led to these different results. In the regression analysis, there were only three instances of significant contribution of standardized measures (out of eight that were included across the three models): the PLS-5 EC for communication and exchange length, and the ADOS-2 SA for communication. The results indicate that for every increase of 10 raw points on the PLS-5 EC, the child’s rate of communication acts would increase by about 7 acts in 10 min. For context, moving from a raw score of 15 to 25 (which is 5 points on either side of the mean raw score in our sample) is the difference between a 10-month to a 20-month age equivalent on the PLS-5 EC. A noteworthy finding is that Expressive Communication was related to children’s communication and exchange length, whereas receptive language was not. This supports prior reports on the importance of language expression for stronger child outcomes (Tager-Flusberg & Kasari, 2013). These results also suggest that giving children the communication systems or tools (e.g., SGDs) and instruction they need to foster expressive language may improve independent communication and length of exchanges with peers. It is important to note that the population for these studies is narrow relative to the broader autism population, but differences in performance on standardized tests may not be critical in determining who may or may not benefit from a blended intervention combining peer-partner instruction and SGDs.

### 4.1. Clinical Implications

A diagnosis of autism is characterized by a wide range of strengths and learning challenges. This heterogeneity leads to marked variability in development and child trajectories in response to interventions (Kim et al., 2018). The purpose of this paper was to determine characteristics associated with peer-directed communication skills following an SGD intervention for a subpopulation of autistic children with limited spoken language. With this knowledge, early service providers and practitioners can make more informed decisions on appropriate social goals, effective intervention approaches, and how to support families of children with complex communication needs. Beukelman and Light (2020) stated the importance of providing specific guidance and developing appropriate goals for individuals using AAC in order for them to develop and grow in language and social competencies. Our findings support the view that for young autistic children, instruction in learning to communicate with a speech-generating device within reciprocal interactions with non-autistic peers may lead to stronger communication skills over time. Specific intervention strategies that focus on Expressive Communication with SGDs may improve peer-directed communication. Children who participate in guided adult-social activities with peers who are comfortable with and understand their AAC system are more likely to be motivated to engage and learn new communication and play skills. Approaches may include providing both communication partners access to the AAC system, supporting turn-taking through any modality (verbal or nonverbal), and setting up the environment for success (e.g., preferred activities that are rewarding for all, limit children in groups, and teach peers responsive skills) (Bourque, 2020). Based on observations of child–peer communicative interactions in naturalistic settings, goals should be tailored to match children’s communication strengths and needs as expressed through any combination of modalities (e.g., SGD, oral speech, signs, gestures, and picture symbols). Although not a focus of this study, it would also be important to determine what peer behaviors and characteristics may impact children’s communication within dyadic interactions, as social reciprocity is dynamic and could be driven or limited by either partner. Outcomes of the current study also indicate that autistic children’s low performance on standardized developmental assessments may not be indicative of how they will benefit or respond to AAC interventions with peer partners, and thus test results should not be viewed as a barrier for participation. Combining direct observational measures from inclusive educational settings with standardized assessments may provide a more comprehensive and ecologically valid picture of children’s communication abilities to inform early intervention planning and goal setting.

### 4.2. Limitations

There are limitations of this study that deserve a mention. First, although the participants were part of an ongoing SMART design study, these analyses were conducted in a single-group format, with all children receiving the SPT with SGD intervention. So, although the statistical analyses showed growth in the targeted social communication outcomes, there was not a control group to rule out other causal factors. Second, we focused on the outcome data after the 10 initial sessions since this occurred prior to re-randomization in the SMART design. Thus, children received only about five weeks of intervention, and predictive relationships may change if looking at outcomes after the full course of intervention (24 sessions). Additionally, the effect sizes were relatively small, but this is potentially expected based on the relatively short intervention and previous research with similar populations. Even so, understanding predictors of early response to a peer-partner intervention is valuable for an understudied population of young autistic children with limited or no spoken language. Finally, there are two potential limitations related to our predictor selection. We used a data-driven approach due to the exploratory nature of the study, which may result in less stable estimates, particularly with a small sample size. Therefore, the results should be considered in light of the exploratory nature. Additionally, there are other potentially predictive factors that were not included in our planned analysis, such as child or family characteristics, peer characteristics or behaviors at baseline and fidelity of implementation.

### 4.3. Future Directions

Although the results of this study provide guidance about potential predictors of outcomes for minimally or non-speaking autistic children engaging in a blended peer-partner communication instruction with SGDs, there are a variety of areas to explore in future research. As noted above, the ongoing SMART design study will include further opportunities after study completion to explore predictors across a larger sample, a longer time, and examine more nuanced features of peer-partner instruction and SGD approaches. While this study focused on child characteristics from naturalistic observations and conventional developmental and language measures, which is important in considering which interventions work for whom, examining other potential predictors may be especially valuable. For example, understanding how previous exposure to SGDs impacts outcomes may help to determine the order of introduction of intervention components (e.g., SGD instruction and peer-partner approaches). Other important child characteristics to consider include baseline engagement, peer imitation skills, and developmental play levels to support children’s early communication and peer interactions. Looking at how child and peer characteristics and behaviors mutually impact each other may provide guidance in recruiting peers and creating activities that all children enjoy. In addition, attending to how the regularity of interactions with peer partners (e.g., in a classroom versus only during intervention sessions) influences child outcomes may have policy implications for schools. Finally, continuing to explore the value of identifying important child-level predictors across various types of early intervention programs will be important to provide recommendations for practice.

## 5. Conclusions

To our knowledge, this study is among the first to report on predictors of communication skills for young autistic children with limited or no speech following participation in an evidence-based social communication intervention with non-autistic peers. Further work is ongoing to determine the causative nature of the effects of SPT with SGD on the participants social communication after a longer period of intervention and random assignment to two groups. At this time, results of the present study show that children’s spontaneous communication significantly improved with peer partners following intervention, an important finding given the complexity of the children’s communication challenges. Furthermore, we identified spontaneous communication, reciprocity, and length of reciprocal exchanges as significant predictors of the same metric after the short peer-partner SGD intervention. Children’s expressive language performance and communication with peers was significantly related to peer-directed communication and reciprocal exchanges, indicating the importance of providing early access to SGDs or other communication tools. Contrary to other reports, children’s receptive language was not significantly correlated with any communication outcomes, warranting further examination. In summary, the results suggest that it may be beneficial for school teams to gather information on how often and for how long autistic children participate in social interactions with peers, with the understanding that fewer attempts to communicate may indicate a need for additional supports.

## Figures and Tables

**Table 1:** Demographic information for autistic participants (*N* = 56).

Assessment	ADOS-2 (Overall Total)	MSEL:ELC (SS)	PLS-5 AC (SS)	PLS-5 EC (SS)	PLS-5 TL (SS)
Mean	19.6	50.2	53.3	55.6	108.9
Range	5–26	49–80	50–89	50–79	100–161

Note. ADOS-2 = Autism Diagnostic Observation Schedule–Second Edition, Module 1 (Lord et al., 2012); MSEL = Mullen Scales of Early Learning (Mullen, 1995); ELC = Early Learning Composite; PLS-5 = Preschool Language Scale–Fifth Edition (Zimmerman et al., 2011); AC = Auditory Comprehension; EC = Expressive Communication; TL = total language; SS = standard score.

**Table 2:** Definitions of communication codes.

Modality	Definition	Examples
Speech	Child uses verbal expressive language that can be clearly understood as a word or words. Must contain at least one vowel sound and either (a) one consonant or (b) one substituted consonant in matching placement of intended word. Must show orientation to other child or to joint activity through body shift and/or gaze, or use of name.	“oh-no”, “wed” for red, “pa” for ball, “my turn”
SGD	Child selects buttons on a speech-generating device (SGD) to intentionally communicate by showing orientation to other child or to joint activity through body shift and/or gaze.	Selects ‘blue’ icon, looks to toy Selects ‘more’ icon, orients
SpeechSGD	Child combines speech and SGD acts, with either modality overlapping or occurring within 3 s of the other. Must show orientation to other child or to joint activity through body shift and/or gaze away from SGD.	Says “fast” and selects car icon Selects ‘my turn’ icons then says “more”
Gesture	Child uses a conventional, culturally recognizable gesture or sign language. Must show orientation to other child or to joint activity through body shift and/or gaze immediately after the gesture.	Gives an object Reach, open hand Shakes head ‘no’
Vocalization	Child vocalizes sound(s) not recognizable as a word with a minimum of one vowel and may include one or more consonants. Must show orientation to other child or to joint activity through body shift and/or gaze. Peer vocalizations were coded as speech.	“ooee” then orients to toy “badada” then orients to peer
Behaviors		
Initiations	Child initiates communication to another child with intentionality using any modality. Initiations are defined by a full 3 s pause after the end of any previous communication act.	
Responses	Child responds to another child’s communicative act using any modality within 3 s. Each consecutive response by either child is coded if child responds again after a full 1 s pause after the last communication act.	
Examples	Peer says “Do you want one?” and 2 s passes, child says “more”; peer says “here you go”—Peer IN + Child RS + Peer RS Child vocalizes “iii” and peer gives toy, then child selects icon for ‘blue’ and 2 s later says “ah”—Child IN + Peer RS + Child RS + Child RS	

Note. SGD = speech-generating device; IN = initiation; RS = response.

**Table 3:** Descriptive statistics for standardized assessments and video-coded communication, reciprocity, and exchange length data.

	Baseline		Post-Treatment		*S*	95% CI
Variables	Mean (SD)	Min–Max	Mean (SD)	Min–Max		
Assessments						
ADOS-2 SA	14.09 (4.00)	2–20	–	–	–	–
ADOS-2 RRB	5.48 (1.67)	1–8	–	–	–	–
PLS-5 AC	21.07 (5.43)	13–39	–	–	–	–
PLS-5 EC	20.16 (5.53)	8–30	–	–	–	–
MSEL VR	22.27 (5.76)	9–42	–	–	–	–
MSEL FM	22.53 (5.75)	8–44	–	–	–	–
Video Data						
Communication	0.84 (0.69)	0.00–3.75	1.93 (1.46)	0.06–6.65	677.50 ***	[0.76–1.44]
Reciprocity	0.30 (0.37)	0.00–1.85	1.25 (1.28)	0.00–5.90	719.50 ***	[0.67–1.23]
Exchange Length	1.02 (0.63)	0.00–4.00	1.56 (0.63)	0.00–4.21	690.50 ***	[0.35–0.72]

Note: *S* = Wilcoxon Signed-Rank Test statistic; CI = confidence interval, reflecting the median difference score on the Wilcoxon Signed-Rank Test; ADOS-2 SA = Autism Diagnostic Observation Schedule–Second Edition Social Affect algorithm raw score; ADOS-2 RRB = Autism Diagnostic Observation Schedule–Second Edition Repetitive and Restrictive Behavior algorithm raw score; PLS-5 AC = Preschool Language Scale–Fifth Edition Auditory Comprehension raw score; PLS-5 EC = Preschool Language Scale–Fifth Edition Expressive Communication raw score; MSEL VR = Mullen Scales for Early Learning Visual Reception raw score; MSEL FM = Mullen Scales for Early Learning Fine Motor raw score. *** *p* < 0.001.

**Table 4:** Correlations of outcome variables with potential predictor variables.

Predictor Variables	Communication Post-Tx	Reciprocity Post-Tx	Exchange Length Post-Tx
ADOS-2 SA	r = −0.44 ***	r = −0.38 **	r = −0.20
ADOS-2 RRB	r = −0.14	r = −0.07	r = −0.01
PLS-5 AC	r = 0.21	r = 0.22	r = 0.14
PLS-5 EC	r = 0.45 ***	r = 0.38 **	r = 0.37 **
MSEL VR	r = 0.32 *	r = 0.34 *	r = 0.31 *
MSEL FM	r = 0.27	r = 0.30 *	r = 0.26
Communication (BL)	r = 0.51 ***	r = 0.46 **	r = 0.34 *
Reciprocity (BL)	r = 0.45 ***	r = 0.50 ***	r = 0.39 **
Exchange Length (BL)	r = 0.19	r = 0.26	r = 0.30 **

Note: Post-Tx = post-treatment; BL = baseline; ADOS-2 SA = Autism Diagnostic Observation Schedule–Second Edition Social Affect algorithm raw score; ADOS-2 RRB = Autism Diagnostic Observation Schedule–Second Edition Repetitive and Restrictive Behavior algorithm raw score; PLS-5 AC = PLS-5 = Preschool Language Scale–Fifth Edition Auditory Comprehension; PLS-5 EC = PLS-5 = Preschool Language Scale–Fifth Edition Expressive Communication; MSEL VR = Mullen Scales for Early Learning Visual Receptions raw score; MSEL FM = Mullen Scales for Early Learning Visual Fine Motor raw score. * *p* < 0.05. ** *p* < 0.01. *** *p* < 0.001.

**Table 5:** Regression model predicting communication post-treatment.

Predictor	*B*	*SE*	β	*t*	*p*	95% CI
Intercept	1.27	1.06	–	1.20	0.230	[−0.86, 3.39]
Communication-BL	0.70	0.24	0.37	2.98	**0.004**	[0.23, 1.17]
ADOS-2 SA	−0.08	0.04	−0.25	−2.03	**0.047**	[−0.16, 0.00]
PLS-5 EC	0.07	0.03	0.30	2.49	**0.016**	[0.01, 0.13]
MSEL VR	−0.01	0.03	−0.05	−0.42	0.680	[−0.07, 0.05]

Note. Model statistics: *R*^2^ = 0.42, adjusted *R*^2^ = 0.37, *F*(4, 50) = 9.06, *p* < 0.0001. *B* = unstandardized coefficient; *SE* = standard error; β = standardized coefficient; CI = confidence interval; values in bold denote significance; BL = baseline; ADOS-2 SA = Autism Diagnostic Observation Schedule–Second Edition Social Affect algorithm score; PLS-5 EC = Preschool Language Scale–Fifth Edition Expressive Communication raw score; MSEL VR = Mullen Scales of Early Learning Visual Reception raw score.

**Table 6:** Regression model for reciprocity post-treatment.

Predictor	*B*	*SE*	β	*t*	*p*	95% CI
Intercept	0.64	0.80	–	0.80	0.430	[−0.97, 2.26]
Reciprocity-BL	1.17	0.31	0.43	3.73	**<0.001**	[0.54, 1.80]
ADOS-2 SA	−0.05	0.03	−0.22	−1.80	0.077	[−0.11, 0.01]
PLS-5 EC	0.04	0.02	0.23	1.96	0.056	[−0.00, 0.09]
MSEL VR	0.00	0.02	0.02	0.18	0.860	[−0.04, 0.05]

Note. Model statistics: *R*^2^ = 0.41, adjusted *R^2^* = 0.37, *F*(4, 50) = 8.84, *p* < 0.0001. *B* = unstandardized coefficient; *SE* = standard error; β = standardized coefficient; CI = confidence interval; values in bold denote significance; BL = baseline; ADOS-2 SA = Autism Diagnostic Observation Schedule–Second Edition Social Affect algorithm score; PLS-5 EC = Preschool Language Scale–Fifth Edition Expressive Communication raw score; MSEL VR = Mullen Scales of Early Learning Visual Reception raw score.

**Table 7:** Regression model for exchange length at post-treatment.

Predictor	*B*	*SE*	β	*t*	*p*	95% CI
Intercept	0.53	0.28	–	1.90	0.060	[−0.03, 1.10]
Exchange Length-BL	0.30	0.11	0.36	2.71	**0.009**	[0.08, 0.51]
Reciprocity-BL	0.27	0.19	0.19	1.42	0.160	[−0.11, 0.64]
PLS-5 EC	0.03	0.01	0.31	2.56	**0.014**	[0.01, 0.05]
MSEL VR	−0.00	0.01	−0.00	−0.01	0.990	[−0.02, 0.02]

Note. Model statistics: *R*^2^ = 0.36, adjusted *R*^2^ = 0.31, *F*(4, 50) = 7.05, *p* < 0.0001. *B* = unstandardized coefficient; *SE* = standard error, β = standardized coefficient, CI = confidence interval; values in bold denote significance; BL = baseline; PLS-5 EC = Preschool Language Scale–Fifth Edition Expressive Communication raw score; MSEL VR = Mullen Scales of Early Learning Visual Reception raw score.

## Data Availability

Data for this study is currently unavailable since the full study is not complete. However, a subset of the data was uploaded to the National Database of Autism Research at the U.S. National Institutes of Health annually.

## Supplementary Materials

The following supporting information can be downloaded at: https://www.mdpi.com/article/10.3390/bs16101962/s1, Table S1: Regression model for communication at 5-weeks with full sample (*N* = 56), Table S2: Regression model for reciprocity at 5-weeks with full sample (*N* = 56), Table S3: Regression model for exchange length at 5-weeks with full sample (*N* = 56).

## References

[B1-behavsci-16-01962] Alzahrani M., Alharbi M., Alodwani A. (2019). The effect of social-emotional competence on children academic achievement and behavioral development. International Education Studies.

[B2-behavsci-16-01962] American Psychiatric Association (2013). Diagnostic and statistical manual of mental disorders.

[B3-behavsci-16-01962] Beukelman D. R., Light J. C. (2020). Augmentative and alternative communication: Supporting children and adults with complex communication needs.

[B4-behavsci-16-01962] Bourque K. (2020). Peer-mediated AAC intervention approaches for young children with ASD and limited to no spoken communication. Perspectives of the ASHA Special Interest Groups.

[B5-behavsci-16-01962] Bourque K., Fleming K., Johner S., Tatli S. (2024). SGD communication intervention for autistic preschoolers: Predictors of response and effectiveness on peer-directed social communication. Technical Research Presentation, American Speech and Hearing Association Conference.

[B6-behavsci-16-01962] Bölte S., Westerwald E., Holtmann M., Freitag C., Poustka F. (2011). Autistic traits and autism spectrum disorders: The clinical validity of two measures presuming a continuum of social communication skills. Journal of Autism and Developmental Disorders.

[B7-behavsci-16-01962] Caudle S. E., Katzenstein J. M., Oghalai J. S., Lin J., Caudle D. D. (2014). Nonverbal cognitive development in children with cochlear implants: Relationship between the Mullen Scales of Early Learning and later performance on the Leiter International Performance Scales–Revised. Assessment.

[B8-behavsci-16-01962] Chen Y. L., Senande L. L., Thorsen M., Patten K. (2021). Peer preferences and characteristics of same-group and cross-group social interactions among autistic and non-autistic adolescents. Autism.

[B9-behavsci-16-01962] Davidson M. M., Ellis Weismer S. (2014). Characterization and prediction of early reading abilities in children on the autism spectrum. Journal of Autism and Developmental Disorders.

[B10-behavsci-16-01962] DiStefano C., Shih W., Kaiser A., Landa R., Kasari C. (2016). Communication growth in minimally verbal children with ASD: The importance of interaction. Autism Research.

[B11-behavsci-16-01962] Eldevik S., Hastings R. P., Hughes J. C., Jahr E., Eikeseth S., Cross S. (2010). Using participant data to extend the evidence base for intensive behavioral intervention for children with autism. American Journal on Intellectual and Developmental Disabilities.

[B12-behavsci-16-01962] English K., Goldstein H., Shafer K., Kaczmarek L. (1997). Promoting interactions among preschoolers with and without disabilities: Effects of a buddy skills-training program. Exceptional Children.

[B13-behavsci-16-01962] Goldstein H., English K., Shafer K., Kaczmarek L. (1997). Interaction among preschoolers with and without disabilities: Effects of across-the-day peer intervention. Journal of Speech, Language, and Hearing Research.

[B14-behavsci-16-01962] Goldstein H., Kaczmarek L., Warren S., Reichle J. (1992). Promoting communicative interaction among children in integrated intervention settings. Causes and effects in communication and language intervention.

[B15-behavsci-16-01962] Hume K., Sam A., Mokrova I., Reszka S., Boyd B. A. (2019). Facilitating social interactions with peers in specialized early childhood settings for young children with ASD. School Psychology Review.

[B16-behavsci-16-01962] JMP Statistical Discovery LLC (2026). JMP *(Student Edition 19.1.0) [Computer software]*.

[B17-behavsci-16-01962] Kaiser A. P., Hampton L. H., McCauley R. J., Fey M. E., Gillam R. (2017). Enhanced milieu teaching. Treatment of language disorders in children.

[B18-behavsci-16-01962] Kasari C., Gulsrud A. C., Shire S. Y., Strawbridge C. (2021a). The JASPER model for children with autism: Promoting joint attention, symbolic play, engagement, and regulation.

[B19-behavsci-16-01962] Kasari C., Kaiser A., Goods K., Nietfeld J., Mathy P., Landa R., Murphy S., Almirall D. (2014). Communication interventions for minimally verbal children with autism: A sequential multiple assignment randomized trial. Journal of the American Academy of Child and Adolescent Psychiatry.

[B20-behavsci-16-01962] Kasari C., Shire S., Shih W., Almirall D. (2021b). Getting SMART about social skills interventions for students with ASD in inclusive classrooms. Exceptional Children.

[B21-behavsci-16-01962] Kasari C., Shire S., Shih W., Landa R., Levato L., Smith T. (2023). Spoken language outcomes in limited language preschoolers with autism and global developmental delay: RCT of early intervention approaches. Autism Research.

[B22-behavsci-16-01962] Kent-Walsh J., McNaughton D. (2005). Communication partner instruction in AAC: Present practices and future directions. Augmentative and Alternative Communication.

[B23-behavsci-16-01962] Kent-Walsh J., Murza K. A., Malani M. D., Binger C. (2015). Effects of communication partner instruction on the communication of individuals using AAC: A meta-analysis. Augmentative and Alternative Communication.

[B24-behavsci-16-01962] Kim S. H., Bal V. H., Benrey N., Choi Y. B., Guthrie W., Colombi C., Lord C. (2018). Variability in autism symptom trajectories using repeated observations from 14 to 36 months of age. Journal of the American Academy of Child & Adolescent Psychiatry.

[B25-behavsci-16-01962] Koegel L. K., Bryan K. M., Su P. L., Vaidya M., Camarata S. (2020). Definitions of nonverbal and minimally verbal in research for autism: A systematic review of the literature. Journal of Autism and Developmental Disorders.

[B26-behavsci-16-01962] Koo T. K., Li M. Y. (2016). A guideline of selecting and reporting intraclass correlation coefficients for reliability research. Journal of Chiropractic Medicine.

[B27-behavsci-16-01962] Laister D., Stammler M., Vivanti G., Holzinger D. (2021). Social-communicative gestures at baseline predict verbal and nonverbal gains for children with autism receiving the Early Start Denver Model. Autism.

[B28-behavsci-16-01962] Locke J., Shih W., Kretzmann M., Kasari C. (2016). Examining playground engagement between elementary school children with and without autism spectrum disorder. Autism.

[B29-behavsci-16-01962] Lord C., Charman T., Havdahl A., Carbone P., Anagnostou E., Boyd B., Carr T., de Vries P. J., Dissanayake C., Divan G., Freitag C. M., Gotelli M. M., Kasari C., Knapp M., Mundy P., Plank A., Scahill L., Servili C., Shattuck P., McCauley J. B. (2022). The Lancet Commission on the future of care and clinical research in autism. The Lancet.

[B30-behavsci-16-01962] Lord C., DiLavore P. C., Gotham K. (2012). Autism diagnostic observation schedule.

[B31-behavsci-16-01962] Mullen E. M. (1995). Mullen scales of early learning.

[B32-behavsci-16-01962] National Autism Center (2015). Findings and conclusions: National standards project, phase 2.

[B33-behavsci-16-01962] Noldus Information Technology (2023). The Observer XT *(Version 17) [Computer software]*.

[B34-behavsci-16-01962] Osborne K., Ledford J. R., Martin J., Thorne K. (2019). Component analysis of stay, play, talk interventions with and without self-monitored group contingencies and recorded reminders. Topics in Early Childhood Special Education.

[B35-behavsci-16-01962] PRC-Saltillo (2026a). LAMP words for life *(Version 2026.2.2) [Mobile app]. Apple App Store*.

[B36-behavsci-16-01962] PRC-Saltillo (2026b). TouchChat HD: AAC with WordPower *(Version 2026.2.0) [Mobile app]. Apple App Store*.

[B37-behavsci-16-01962] Privitera R., Piccolo A., De Domenico C., Leonardi G., Alito A., Quartarone A., Cucinotta F., Di Cara M. (2026). Interventions to promote communication in minimally verbal autistic children: A systematic review. Medical Sciences.

[B38-behavsci-16-01962] Romski M., Sevcik R. A., Barton-Hulsey A., Whitmore A. S. (2015). Early intervention and AAC: What a difference 30 years makes. Augmentative and Alternative Communication.

[B39-behavsci-16-01962] Saban-Bezalel R., Ben-Itzchak E., Zachor D. A. (2025). Friendship in autism spectrum disorder is related to diverse developmental changes between toddlerhood and adolescence. Journal of Autism and Developmental Disorders.

[B40-behavsci-16-01962] Schlosser R. W., Koul R. K. (2015). Speech output technologies in interventions for individuals with autism spectrum disorders: A scoping review. Augmentative and Alternative Communication.

[B41-behavsci-16-01962] Severini K. E., Ledford J. R., Barton E. E., Osborne K. C. (2019). Implementing stay-play-talk with children who use AAC. Topics in Early Childhood Special Education.

[B42-behavsci-16-01962] Smith T., Iadarola S. (2015). Evidence base update for autism spectrum disorder. Journal of Clinical Child & Adolescent Psychology.

[B43-behavsci-16-01962] Tager-Flusberg H., Kasari C. (2013). Minimally verbal school-aged children with autism spectrum disorder: The neglected end of the spectrum. Autism Research: Official Journal of the International Society for Autism.

[B44-behavsci-16-01962] Therrien M. C., Light J., Pope L. (2016). Systematic review of the effects of interventions to promote peer interactions for children who use aided AAC. Augmentative and Alternative Communication.

[B45-behavsci-16-01962] Thiemann-Bourque K., Feldmiller S., Hoffman L., Johner S. (2018). Incorporating a peer-mediated approach into speech-generating device intervention: Effects on communication of preschoolers with autism spectrum disorder. Journal of Speech, Language, and Hearing Research.

[B46-behavsci-16-01962] Thiemann-Bourque K., McGuff S., Goldstein H. (2017). Training peer partners to use a speech-generating device with classmates with autism spectrum disorder: Exploring communication outcomes across preschool contexts. Journal of Speech, Language, and Hearing Research.

[B47-behavsci-16-01962] Vivanti G., Prior M., Williams K., Dissanayake C. (2014). Predictors of outcomes in autism early intervention: Why don’t we know more?. Frontiers in Pediatrics.

[B48-behavsci-16-01962] Warreyn P., Van der Paelt S., Roeyers H. (2014). Social-communicative abilities as treatment goals for preschool children with autism spectrum disorder: The importance of imitation, joint attention, and play. Developmental Medicine & Child Neurology.

[B49-behavsci-16-01962] Wendt O. (2024). Invited commentary: Current trends in augmentative and alternative communication intervention for minimally verbal autistic children. Revista Psicologia: Teoria e Prática.

[B50-behavsci-16-01962] Zimmerman I. L., Steiner V. G., Pond R. E. (2011). Preschool language scales—fifth edition (PLS-5).

