# Supplementary material for: Predictors of Communication Skills Following a Peer Partner Intervention for Young Autistic Children with Limited or No Spoken Language
Source: Behav Sci (Basel). 2026 Oct 21;16(10):1962. doi: 10.3390/bs16101962 (PMC13701253; doi:10.3390/bs16101962)
Supplement: Supplementary file 1 [file behavsci-16-01962-s001.zip › behavsci-4524800-supplementary.pdf]

Supplemental Materials

Table S1. Regression model for communication at 5–weeks with full sample (*N*=56).

**Predictor**	***B***	***SE***	***β***	***t***	***p***	**95% CI**
Intercept	0.98	1.18	--	0.83	.41	[-1.39, 3.37]
Communication–BL	0.72	0.26	0.34	2.75	**.008**	[0.19, 1.25]
ADOS–2 SA	-0.08	0.04	-0.22	-1.83	.073	[-0.17, 0.01]
PLS–5 EC	0.08	0.03	0.29	2.45	**.017**	[0.01, 0.14]
Mullen VR	-0.00	0.03	-0.01	-0.10	.92	[-0.06, 0.06]

Model statistics: $R^2 = 0.40$, adjusted $R^2 = 0.35$, $F_{(4, 51)} = 8.51$, $p < .0001$

Caption: The table includes output for a regression model for the outcome of communication and predictors of baseline communication, ADOS–2 Social Affect algorithm score, PLS–5 Expressive Communication raw score, and Mullen Visual Reception raw score with the full sample. The table in the manuscript excludes one participant who was an outlier.

Note. *B* = unstandardized coefficient; *SE* = standard error; *β* = standardized coefficient; CI = confidence interval; bold values denote significance; BL = baseline; ADOS–2 SA = Autism Diagnostic Observation Schedule – Second Edition Social Affect algorithm score (Lord et al., 2012); PLS–5 EC = Preschool Language Scale – Fifth Edition Expressive Communication raw score (Zimmerman et al., 2011); Mullen VR = Mullen Scales of Early Learning Visual Reception raw score (Mullen, 1995).

Table S2. Regression model for reciprocity at 5–weeks with full sample (*N*=56).

**Predictor**	***B***	***SE***	***β***	***t***	***p***	**95% CI**
Intercept	0.36	0.97	--	0.37	.71	[-1.59, 2.31]
Reciprocity–BL	1.24	0.38	0.39	3.26	**.002**	[0.48, 2.00]
ADOS–2 SA	-0.05	0.04	-0.18	-1.50	.14	[-0.13, 0.02]
PLS–5 EC	0.05	0.03	0.23	1.90	.063	[-0.00, 0.10]
Mullen VR	0.01	0.03	0.06	0.47	.64	[-0.04, 0.07]

Model statistics: $R^2 = 0.37$, adjusted $R^2 = 0.32$, $F_{(4, 51)} = 7.58$, $p < .0001$

Caption: The table includes output for a regression model for the outcome of reciprocity and predictors of baseline reciprocity, ADOS–2 Social Affect algorithm score, PLS–5 Expressive Communication raw score, and Mullen Visual Reception raw score with the full sample. The table in the manuscript excludes one participant who was an outlier.

Note. *B* = unstandardized coefficient; *SE* = standard error; *β* = standardized coefficient; CI = confidence interval; bold value denote significance; BL = baseline; ADOS–2 SA = Autism Diagnostic Observation Schedule – Second Edition Social Affect algorithm score (Lord et al., 2012); PLS–5 EC = Preschool Language Scale – Fifth Edition Expressive Communication raw score (Zimmerman et al., 2011); Mullen VR = Mullen Scales of Early Learning Visual Reception raw score (Mullen, 1995).

Table S3. Regression model for exchange length at 5–weeks with full sample (*N*=56).

**Predictor**	***B***	***SE***	***β***	***t***	***p***	**95% CI**
Intercept	0.37	0.35	--	1.06	.29	[-0.33, 1.08]

Exchange Length–BL	0.27	0.14	0.27	1.95	.057	[-0.01, 0.54]
Reciprocity–BL	0.33	0.23	0.19	1.41	.17	[-0.14, 0.80]
PLS–5 EC	0.03	0.01	0.30	2.33	**.024**	[0.00, 0.06]
Mullen VR	0.01	0.01	0.05	0.39	.70	[-0.02, 0.04]

Model statistics: $R^2 = 0.31$, adjusted $R^2 = 0.25$, $F_{(4, 51)} = 5.60$, $p < .001$

Caption: The table includes output for a regression model for the outcome of persistence and predictors of baseline persistence, baseline reciprocity, PLS–5 Expressive Communication raw score, and Mullen Visual Reception raw score with the full sample. The table in the manuscript excludes one participant who was an outlier.

Note. *B* = unstandardized coefficient; *SE* = standard error; *β* = standardized coefficient; CI = confidence interval; bold values denote significance; BL = baseline; ADOS–2 SA = Autism Diagnostic Observation Schedule – Second Edition Social Affect algorithm score (Lord et al., 2012); PLS–5 EC = Preschool Language Scale – Fifth Edition Expressive Communication raw score (Zimmerman et al., 2011); Mullen VR = Mullen Scales of Early Learning Visual Reception raw score (Mullen, 1995).
